# Repeated Clinical Assessment Using Sensory Modality Assessment and Rehabilitation Technique for Diagnosis in Prolonged Disorders of Consciousness

**DOI:** 10.3389/fnhum.2021.728637

**Published:** 2021-12-01

**Authors:** Liliana da Conceição Teixeira, Danielle Blacker, Carlos Campos, Carolina Garrett, Sophie Duport, Nuno Barbosa Rocha

**Affiliations:** ^1^Center for Innovative Care and Health Technology, School of Health Sciences, Polytechnic of Leiria, Leiria, Portugal; ^2^Center for Rehabilitation Research, School of Health, Polytechnic Institute of Porto, Porto, Portugal; ^3^Faculty of Medicine, University of Porto, Porto, Portugal; ^4^Occupational Therapy Department, Royal Hospital for Neuro-Disability, London, United Kingdom; ^5^Laboratory of Neuropsychophysiology, Faculty of Psychology and Educational Sciences, University of Porto, Porto, Portugal; ^6^Research Department, Royal Hospital for Neuro-Disability, London, United Kingdom

**Keywords:** assessment, diagnosis, disorders of consciousness, minimally conscious state, vegetative state (unresponsive wakefulness syndrome)

## Abstract

**Purpose:** The recommended way to assess consciousness in prolonged disorders of consciousness is to observe the patient’s responses to sensory stimulation. Multiple assessment sessions have to be completed in order to reach a correct diagnosis. There is, however, a lack of data on how many sessions are sufficient for validity and reliability. The aim of this study was to identify the number of Sensory Modality Assessment and Rehabilitation Technique (SMART) assessment sessions needed to reach a reliable diagnosis. A secondary objective was to identify which sensory stimulation modalities are more useful to reach a diagnosis.

**Materials and Methods:** A retrospective analysis of all the adult patients (who received a SMART assessment) admitted to a specialist brain injury unit over the course of 4 years was conducted (*n* = 35). An independent rater analyzed the SMART levels for each modality and session and provided a suggestive diagnosis based on the highest SMART level per session.

**Results:** For the vast majority of patients between 5 and 6 sessions was sufficient to reach the final clinical diagnosis. The visual, auditory, tactile, and motor function modalities were found to be more associated with the final diagnosis than the olfactory and gustatory modalities.

**Conclusion:** These findings provide for the first time a rationale for optimizing the time spent on assessing patients using SMART.

## Introduction

The term prolonged disorder of consciousness (PDoC) is used to describe a spectrum of disorders in which consciousness is altered in a transient or permanent way (Monti, 2015). It represents a continuous spectrum of awareness from coma, vegetative state (VS) to minimally conscious state (MCS; Royal College of Physicians, 2020). It describes any disorder of consciousness that has continued for at least 4 weeks following sudden onset brain injury (Royal College of Physicians, 2020). People in a coma have no voluntary behaviors, fail to respond normally to painful stimuli, sound or light and their eyes remain closed. After coma, a person may regain full consciousness, or evolve into VS or MCS. A person in VS is unable to show meaningful responses to any stimuli, however, a range of reflexive and spontaneous behaviors may be preserved (Royal College of Physicians, 2020). MCS patients show inconsistent but discernible signs of behavioral activity that is more than reflexive activity (Giacino et al., 2002). Patients in MCS show some evidence of self and environmental awareness (Giacino and Kalmar, 2005; Bernat, 2006). MCS patients are further subcategorized based on the complexity of their behaviors. The term MCS-minus is used to describe patients with low-level behavioral responses (i.e., patients who can demonstrate visual pursuit, localize noxious stimulation or contingent behaviors such as appropriate smiling or crying to emotional stimuli). MCS-plus is used to describe patients with high-level behavioral responses, such as command following, intelligible verbalization and intentional communication (Bruno et al., 2011; Thibaut et al., 2020).

Patients in PDoC are vulnerable to misdiagnosis that can negatively affect their rehabilitation process (Gosseries et al., 2011). Differentiating VS from MCS is challenging since purposeful, spontaneous and reflexive behaviors can be difficult to differentiate and subtle signs of consciousness may be missed. Several studies have shown that misdiagnosis of VS and MCS patients is common (Andrews et al., 1996; Childs and Mercer, 1996; Gill-Thwaites and Munday, 2004; Schnakers et al., 2009) with possible implications on decisions relating to continuation of life support, indication for neurorehabilitation, caretaker planning and family adjustment (Hirschberg and Giacino, 2011; da Conceição Teixeira et al., 2021). Moreover, consciousness may fluctuate over time and behaviors that might indicate higher levels of consciousness are exhibited episodically and intermittently (i.e., in the MCS patient) making diagnosis more challenging and increasing potential for error (Fins, 2016). Furthermore, this classic terminology was devised before the development of techniques such as functional magnetic resonance (fMRI) and it does not take into consideration the patient’s overt and covert consciousness (da Conceição Teixeira et al., 2021). Several studies report that when VS patients are assessed using fMRI a significant number are indeed conscious (Owen et al., 2006; Monti et al., 2010; Naci et al., 2014). However, diagnosis of people in VS or MCS still depend on the assessment of responses to stimulation (Bodart et al., 2013) while functional assessment by neuroimaging remains mostly limited to research (Cortese et al., 2015).

It is widely recognized that the use of standardized and sensitive behavioral assessment scales can help clinicians to identify subtle signs of consciousness (Heine et al., 2013). A systematic review of behavioral assessment scales of PDoC conducted by Seel et al. (2010), identified 13 instruments, six of which could be used with minor to moderate reservations to assess PDoC. Three of them are commonly used by clinicians working in PDoC: the JFK Coma Recovery Scale – Revised (CRS-R); the Wessex Head Injury Matrix (WHIM); and the Sensory Modality Assessment and Rehabilitation Technique (SMART; Royal College of Physicians, 2020). The CRS-R has been identified as the most sensitive scale and can be used with minor reservation in the assessment of PDoC, whilst SMART and WHIM can be used with moderate reservations to assess this population (Seel et al., 2010). The Royal College of Physicians (RCP) guidelines recommends the use of one or more of these three instruments for formal structured assessment of PDoC (Royal College of Physicians, 2020).

Regardless of the assessment tool used, diagnosis cannot be based on a single assessment. Since consistency of behaviors is the key to accurate diagnosis; it is important that observations are repeated and reviewed over time using a combination of detailed clinical evaluation and validated, structured assessment tools. This will ensure that potential awareness and any fluctuations in awareness are identified and interpreted accurately (Owen and Coleman, 2008; Cortese et al., 2015; Royal College of Physicians, 2020). A recent study suggested that at least five assessment sessions using the CRS-R should be conducted within a period of 2 weeks to reduce misdiagnosis (Wannez et al., 2017). SMART currently recommends a series of 10-session assessment within a 2–3 week period. This systematic observation provides quantitative information of the frequency and quality of responses and will assist the assessor in the diagnostic process (Gill-Thwaites and Munday, 2004). However, serial assessments are a heavy burden for the patient and for the clinical team and should not be done as a matter of routine (Royal College of Physicians, 2020). Patients at the early stage of rehabilitation may have limited sitting tolerance meaning that they can only sit for a few hours per day, hence limiting their therapy and assessment time. Patients are easily fatigued and care should be taken not to overburden them. It is therefore extremely important to quantify the minimum number of sessions required to reach an accurate diagnosis to optimize the patient’s participation in rehabilitation and to increase cost efficiency.

The main goal of this study is to evaluate SMART diagnosis reliability across the assessment sessions. This will allow the development of recommendations regarding the minimum number of sessions required to reach a reliable diagnosis. Furthermore, this study also aims to identify which SMART modalities contribute to the diagnosis.

## Materials and Methods

### Assessment Tool

Sensory Modality Assessment and Rehabilitation Technique is both an assessment and treatment tool designed to elicit behavioral responses to a comprehensive range of stimuli. SMART is divided into formal and informal assessment. The SMART informal component investigates the family and the team’s observation of responses over time. The SMART formal component comprises two parts: the SMART Behavioral Assessment and the SMART Sensory Assessment (Gill-Thwaites and Munday, 2004). In this study we will focus on the latter.

Sensory Modality Assessment and Rehabilitation Technique Sensory Assessment is composed of 29 standardized techniques distributed across eight modalities, including five sensory (visual, auditory, tactile, olfactory, and gustatory) and three other modalities of motor function, functional communication, and wakefulness/arousal (Gill-Thwaites, 1997). This assessment contains sensory stimulation techniques, including, for example, response to light, visual tracking, and following specific written or verbal instructions. These are designed to elicit behavioral responses arranged in a hierarchical way. The highest-level responses represent the most complex behaviors for that modality. Responses in each modality are assessed on a five-point hierarchical scale. Findings are summarized in terms of the SMART levels (1 – no response; 2 – reflexive response; 3 – withdrawal response; 4 – localizing response; and 5 – differentiating response; Royal College of Physicians, 2020). Response levels between 1 and 3 are indicative of a VS diagnosis, whilst responses at levels 4 or 5 are indicative of MCS-minus and MCS-plus diagnosis, respectively.

The suggestive diagnosis provided by SMART is based on the presence or absence of these responses to specific stimuli. SMART recommends the assessor conducts 10 sessions within a 3-week period. Frequent assessment enables the assessor to establish if the behavioral responses observed are consistent and repeatable.

### Procedures

A retrospective analysis of the medical notes of all the patients admitted to the Brain Injury Unit at the Royal Hospital for Neuro-disability (RHN) over the course of 4 years (January 2015 to December 2018) was conducted. Inclusion criteria were adult patients with a first episode of brain injury presenting in PDoC. Of those in PDoC, all patients who had the SMART assessment conducted were included. Files were excluded if SMART forms or reports were missing.

Sensory Modality Assessment and Rehabilitation Technique form D records the observed responses to the sensory techniques for all modalities (visual, auditory, tactile, olfactory, gustatory, motor function, functional communication, and wakefulness) over 10 consecutive sessions. For each session the highest-level response for each modality is identified by the assessor and a final diagnosis is concluded at the end of the 10 sessions.

In this study, one independent rater, with over 10 years’ experience working with this population, reviewed SMART form D (10 consecutive SMART session results conducted by a SMART assessor) for each patient. The independent rater provided a suggestive diagnosis per session and a final overall diagnosis based on the highest SMART level observed. The rater was blinded to the final diagnosis (i.e., clinical diagnosis provided by the SMART assessor) of the participants throughout and was provided with data session by session, without knowledge of the next session’s level of response.

Patients were classified in VS or MCS on the final diagnosis by the ward SMART assessors. At the time of data collection, the MCS-minus and MCS-plus definitions were not consistently used terminologies. However, in this study, the rater has provided a diagnosis of VS (highest SMART levels between 1 and 3), MCS-minus (highest SMART level: 4), and MCS-plus (highest SMART level: 5) as per current RCP guidelines (Royal College of Physicians, 2020).

The rater also recorded a percentage score of confidence in their suggestive diagnoses after each session and therefore the number of sessions required to reach a status of 100% confidence in the diagnosis was also computed for each participant. Only one rater was used in this study due to SMART’s excellent inter-rater reliability (Gill-Thwaites and Munday, 2004). The aim of this study was not to test the reliability of the tool but to assess how many sessions were required to reach a diagnosis.

This retrospective analysis was based on the existing records of included patients and this data was always kept and processed anonymously. Data was collected from the patient’s records and were anonymized before being shared with the research team. The ethical principles of the Declaration of Helsinki by the World Medical Association concerning human experimentation were followed (World Medical Association, 2013).

### Data Analysis

Missing values are somewhat common in SMART sessions. Patients may not be assessed, for example, for some of the visual modality techniques due to low arousal levels or gustatory modality techniques may have been omitted due to risk of aspiration. Thus, before proceeding with data analysis, missing values for each subject were replaced by the score of the previous session using the last observation carried forward imputation method. If there was no previous score available (e.g., missing value on session 1), the score of the subsequent session was used. Only participants with at least five scored sessions of any given subscale were included in the analyses for that modality. This number of sessions was chosen based on a recent paper by Wannez et al. (2017), where it is suggested by the authors to perform at least five CRS-r assessment sessions when assessing PDoC patients, to avoid misdiagnosis.

Descriptive statistics such as absolute and relative frequencies were computed for categorical variables. For continuous variables, mean and standard deviation were calculated. Hypothesis testing was used to compare VS and MCS groups regarding sex, etiology, age, time of assessment and number of sessions required to achieve diagnosis. Before proceeding for inferential analysis, assumptions for each procedure were tested. The normality assumption was evaluated by examining skewness and kurtosis values (Zumbo and Jennings, 2002; Schmider et al., 2010; Gignac, 2019) and the homogeneity of variance was tested using Levene’s test (Nordstokke and Zumbo, 2010; Nordstokke et al., 2011). For continuous variables, if assumptions were met, inferential testing was conducted using parametric tests such Student’s *t* test; otherwise, non-parametric alternatives were used (Mann–Whitney *U* test). For binary nominal variables, Fisher’s exact test was applied. Additionally, mixed ANOVA models were explored to determine whether changes in SMART modality level scores is the result of the interaction between diagnosis group (VS and MCS) and the session number (time).

The agreement between each session diagnosis and the final diagnosis was evaluated using agreement percentage and Cohen’s Kappa. Kappa should be interpreted as follows: values ≤ 0 indicate no agreement and 0.01–0.20 none to slight, 0.21–0.40 as fair, 0.41–0.60 as moderate, 0.61–0.80 as substantial, and 0.81–1.00 as almost perfect agreement (McHugh, 2012).

Data analysis was conducted using IBM SPSS (version 26). For all inferential testing procedures, the significance level was set at 0.05.

## Results

Fifty-two PDoC patients were admitted to the brain injury unit at the RHN from January 2015 to December 2018. The clinical notes of 17 patients were excluded due to incomplete data set. The characterization of the study participants is presented in Table 1. The clinical notes of 35 patients with diagnosis of VS (*n* = 10, 28.6%) or MCS (*n* = 25; 71.4%) were analyzed. Patients had a mean [±standard deviation (SD)] age of 49.7 (±15.3) years old, and were mostly male (*n* = 28, 80%). Time from brain injury to SMART assessment spanned from 3 to 70 months with a mean value of 8.2 (±11.1) months. Twenty-four participants had sustained a non-traumatic brain injury and 11 sustained a traumatic brain injury.

**TABLE 1:** Sample characterization (*n* = 35).

	VS (*n* = 10)	MCS (*n* = 25)	*p*-value
**Sex, *n* (%)**			
*Female*	2 (20.0)	5 (20.0)	0.690 ^§^
*Male*	8 (80.0)	20 (80.0)	
**Etiology, *n* (%)**			
*Traumatic brain injury*	3 (30)	8	0.620 ^§^
*Non-traumatic brain injury*	7	17	
**Age (years)**			
Mean ± SD	41.8 ± 16.8	52.9 ± 13.9	0.051 ^†^
**Time of assessment (months)**			
Mean ± SD	6.6 ± 3.6	8.8 ± 12.9	0.706 ^‡^

*Abbreviations: VS, Vegetative State; MCS, Minimally Conscious State; and SD, Standard Deviation.*

*^§^Fisher’s Exact Test; ^†^Independent samples t-test; and ^‡^Mann–Whitney U test.*

Comparison of VS and MCS groups regarding sociodemographic variables (sex, etiology, age, and time of assessment) was conducted. There were no significant differences between groups regarding sex, etiology and time of assessment (*p* > 0.05). There was a marginally significant difference in age between VS (41.8 ± 16.8) and MCS (52.9 ± 13.8) participants (*p* = 0.051).

### Comparison of SMART Levels Per Modality Between Diagnostic Groups Throughout 10 Session-Assessment

Mixed ANOVA models were used to determine whether there were any changes in SMART modality level scores across sessions and between diagnosis groups (VS and MCS). The results showed that there was no significant main effect of the session number on any of the SMART modality level scores. In addition, there was a significant main effect of diagnosis group on the following SMART modality level scores: visual, auditory, tactile, motor function, and functional communication (*p* < 0.05). However, no significant interaction was found between diagnosis group and session number in terms of the SMART modality level scores. Nonetheless, as the normality and homogeneity assumptions were not met for every level of the mixed ANOVA models, the reported results should be interpreted with caution.

**FIGURE 1:**
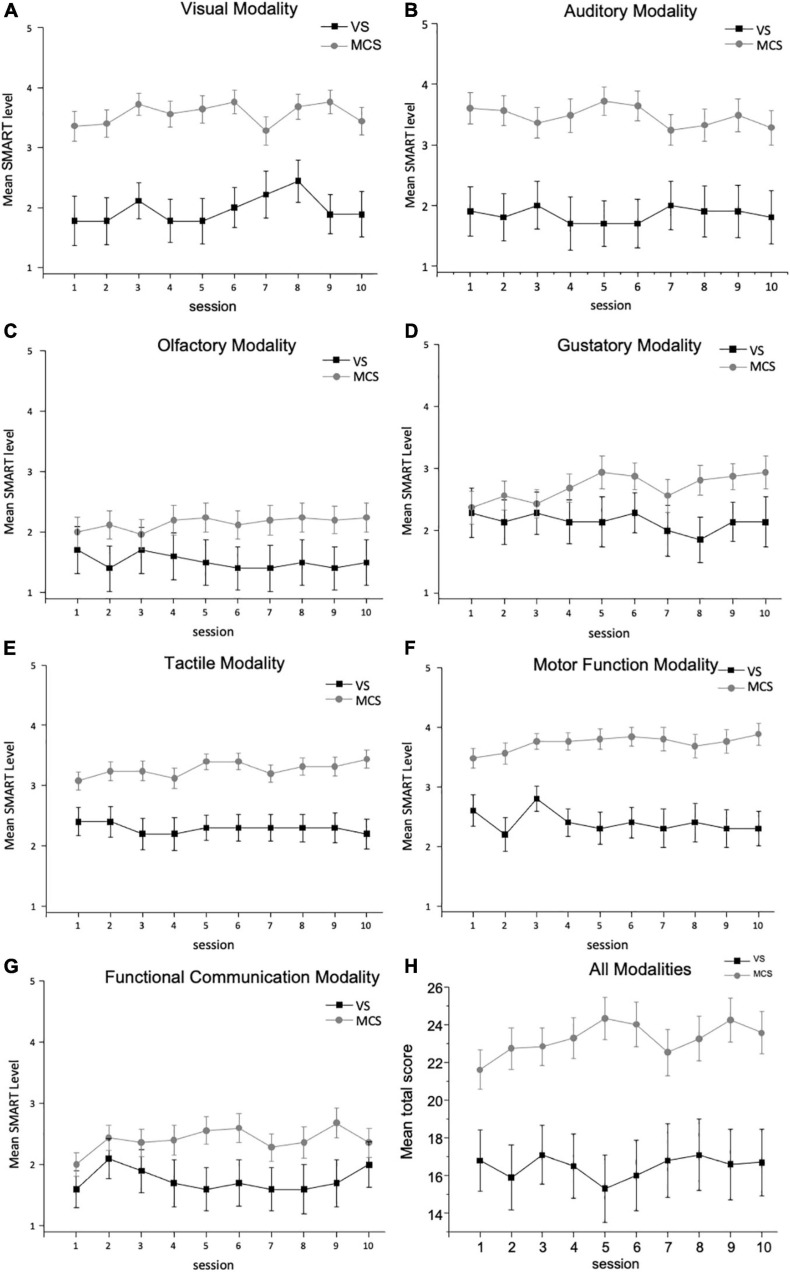
Mean SMART level per diagnosis group ± SEM: **(A)** mean SMART level for VS and MCS groups for the visual modality ± SEM; **(B)** mean SMART level for VS and MCS groups for the auditory modality ± SEM; **(C)** mean SMART level for VS and MCS groups for the olfactory modality ± SEM; **(D)** mean SMART level for VS and MCS groups for the gustatory modality ± SEM; **(E)** mean SMART level for VS and MCS groups for the tactile modality ± SEM; **(F)** mean SMART level for VS and MCS groups for the motor function modality ± SEM; **(G)** mean SMART level for VS and MCS groups for the functional communication modality ± SEM; and **(H)** mean SMART level for VS and MCS groups for total score of all modalities ± SEM.

Figure 1 describes the mean SMART modality level scores throughout the 10 sessions assessment for each modality and for all the modalities for the two diagnosis groups. There is a diagnosis group effect in all modalities, with the MCS group combined having a higher mean score than the VS group as expected, except for the olfactory and gustatory modalities (*p* = 0.071 and *p* = 0.106, respectively). There was no change in the results when age was added as a covariable.

### Confidence to Reach a Diagnosis Throughout the 10-Sessions

On average, 5.66 ± 2.01 sessions were required to reach a diagnosis. A significant difference was found in the number of sessions required to achieve diagnosis for the VS group (6.0 ± 0.0) and the MCS group (5.52 ± 2.38; *p* = 0.017). Although this difference was statistically significant, it took on average six sessions to reach a diagnosis. A significant difference was also found when the number of sessions needed to reach a diagnosis was studied across the group of patients diagnosed by the independent rater with VS (6.0 ± 0.0), MCS-minus (6.91 ± 2.55), and MCS-plus (4.43 ± 1.60; *p* < 0.001). Results show that the number of sessions needed to reach a suggestive diagnosis in the MCS-minus group is significantly greater than in the MCS-plus group (*p* = 0.006).

### Comparison of Session Diagnosis With Final Diagnosis

Table 2 summarizes the agreement between the rater’s diagnosis at each session with the final (actual) diagnosis provided in the clinical records. Overall, percentage agreement and Cohen’s Kappa values are high from the first session onward. The rater’s diagnosis becomes consistently the same after session 3. For the VS group, the rater’s final diagnosis is maintained throughout the 10 assessment sessions.

**TABLE 2:** Agreement evaluation between the diagnosis in each session and the final diagnosis (*n* = 35).

Session	Session/Final evaluation (n)		Total	
	VS/VS (*n* = 10)	MCS/MCS (*n* = 25)	% Agreement	Kappa
1	10	19	82.86	0.64
2	10	21	88.57	0.75
3	10	25	100	1
4	10	25	100	1
5	10	25	100	1
6	10	25	100	1
7	10	25	100	1
8	9	26	97.14	0.93
9	9	26	97.14	0.93
10	9	26	97.14	0.93

From this data we can also determine that were the assessment to stop after the first session approximately 17% of patients would not be diagnosed correctly. This number decreases to 11% after the second assessment session, confirming the importance of continued assessment.

## Discussion

It is important to optimize the execution of consciousness assessments. Clinical management of PDoC patients from treatment pathways to end of life decisions depend on accurate and thorough behavioral observations.

According to SMART, response levels between 1 and 3 (1- no response; 2- reflexive response; and 3- withdrawal response), are indicative of VS diagnosis. If a patient scores a level 4 or 5 (4- localizing response; 5- differentiating response) it is an indication of an MCS diagnosis (Gill-Thwaites and Munday, 2004). Hence, it is expected that VS patients will present with lower scores overall. In this study, it was found that MCS patients have consistently higher SMART level scores than VS patients in most SMART modalities (except for the olfactory and gustatory modalities). Furthermore, the results of this study may indicate that some sensory modalities seem not to influence the diagnosis. These results are in line with a recent study that reported that patients were mostly likely to score early on the wakefulness, auditory and visual modalities and were least likely to pick up early points on the tactile, olfactory and gustatory modalities (Tennant and Gill-Thwaites, 2017). In another study, the motor function modality of the SMART assessment has proven ability to predict a final diagnosis of MCS with high sensitivity and specificity (Kempny et al., 2012). The results from this study may indicate that the techniques used in the visual, auditory, tactile, motor function and functional communication modalities of the SMART assessment may have more predictive value when it comes to final diagnosis. SMART Tracker is a screening tool currently being developed which includes six techniques used in the SMART assessment. Of these six techniques, two are in the visual modality, three in the auditory modality and one in the functional communication modality of SMART (1. Visual focusing on photograph; 2 visual tracking of assessor; 3 responses to voice; 4 following verbal instructions; 5 ability to use auditory switch following verbal instruction; and 6 providing a yes/no response; Tennant and Gill-Thwaites, 2017). It would be important to consider future research on the clinical utility of this short form of the SMART assessment.

Sensory Modality Assessment and Rehabilitation Technique recommends 10 session-assessment over a period of 2–3 weeks (Gill-Thwaites and Munday, 2004). In this study, for the majority of patients 5–6 sessions seem to be sufficient to get to a correct diagnosis. This is in line with a recent study conducted by Wannez et al. (2017), where it is suggested that at least five assessment sessions, using CRS-R, should be conducted within a period of 2 weeks to reduce misdiagnosis (Wannez et al., 2017).

Although it took the independent rater around 5–6 sessions on average to achieve a total confidence status about the diagnosis; it required less sessions to achieve total confidence in diagnosis for MCS-plus participants. The RCP guidelines recommend that multiple assessments are justified in the context of continuing clinical uncertainty but should not be done as a matter of routine. In fact, this study demonstrated that it took the rater the longest time to be certain of the diagnosis when participants were in the lower end of MCS. This is in line with a previous study that reported that it is more common for patients in MCS to be assigned to an uncertain diagnosis (Schnakers et al., 2009). For the VS and MCS-plus diagnostic certainty was achieved earlier on in the assessment.

The independent rater diagnosis varies in the first 3 sessions and after that, changes are minimal. This is in line with a study conducted by Riganello et al. (2015), that shows that fluctuation is particularly common early in the course of recovery. This might be due to the fact that a patient may fluctuate over the course of the assessment but also that it may take some time for the assessor to become familiar with the patient and his/her behavioral repertoire.

Sensory Modality Assessment and Rehabilitation Technique is a highly reliable tool for assessment of PDoC patients. Its stringent training and accreditation program ensure that SMART is consistently applied by trained assessors. Its high interrater reliability and accreditation program may have contributed to this study results. SMART is only conducted by a trained assessor and the independent rater was an experienced SMART assessor. Maybe, for less experienced and non-trained assessors, more sessions may be needed in order to reach a correct diagnosis. SMART is an investigative tool not only used to assess consciousness but also to identify reproducibility of responses through a thorough investigation using core, advanced and explorative techniques as required. The more time spent with the patient, the better they know his/her behavior repertoire and increase opportunities to explore their full potential. Additional time with the patient allows the assessor to create bespoke interventions and management programs to ascertain if the SMART level or frequency of responses can be enhanced. There are limitations to this study: sample size is limited due to low numbers of PDoC patients; participants were not classified into MCS-minus and MCS-plus by the person who assessed and wrote the initial report after conducting SMART assessment as data was collected before the consistent use of this terminology; some data was missing; the descriptive nature of the results and all the limitations associated with a retrospective study. Moreover, the fact that there was no follow-up and there might have been changes in the patients after being discharged. For all these reasons, the results should be interpreted carefully. Nonetheless, this exploratory analysis revealed that for the vast majority of PDoC patients, 5–6 assessment sessions are sufficient to reach an accurate diagnosis protecting clinical resource management. Furthermore, this analysis demonstrated that some modalities have more predictive value than others. Whilst those modalities may not contribute to the diagnosis, they are important to develop a personalized treatment program for the patient. With this in mind, the time spent conducting assessment with PDoC using SMART may decrease significantly. However, a prospective research study is needed to confirm these results.

A machine learning approach could help with diagnosis accuracy and support decision making for this population. Future research is also required to establish which sensory modalities are sufficient to reach a correct diagnosis and to check which techniques are more predictive of the diagnosis.

## Data Availability Statement

The raw data supporting the conclusions of this article will be made available by the authors, without undue reservation.

## Ethics Statement

Ethical review and approval was not required for the study on human participants in accordance with the local legislation and institutional requirements. Written informed consent for participation was not required for this study in accordance with the national legislation and the institutional requirements.

## Author Contributions

LCT, SD, and NR contributed to the conception and study design. LCT and CC analyzed the data. LCT drafted the manuscript. DB, SD, CG, and NR contributed with major revisions. All authors critically reviewed the manuscript and contributed to the editing of the final draft.

## Conflict of Interest

The authors declare that the research was conducted in the absence of any commercial or financial relationships that could be construed as a potential conflict of interest.

## Publisher’s Note

All claims expressed in this article are solely those of the authors and do not necessarily represent those of their affiliated organizations, or those of the publisher, the editors and the reviewers. Any product that may be evaluated in this article, or claim that may be made by its manufacturer, is not guaranteed or endorsed by the publisher.
